# Can different information channels promote farmers’ adoption of Agricultural Green Production Technologies? Empirical insights from Sichuan Province

**DOI:** 10.1371/journal.pone.0308398

**Published:** 2024-08-08

**Authors:** Ruyu Zhang, Yanan Feng, Yufeng Li, Ke Zheng

**Affiliations:** 1 College of Management, Sichuan Agricultural University, Chengdu, Sichuan, China; 2 Department of Politics and International Relations, University of Southampton, Southampton, United Kingdom; Huanggang Normal University, CHINA

## Abstract

Information accessibility is a pivotal factor influencing farmers’ adoption of Agricultural Green Production Technologies (AGPT). However, the widespread issue of information poverty presents a significant obstacle to this adoption process, thereby hindering the progression towards sustainable agricultural development. To address this information deficit, farmers have begun to utilize the Internet and participate in government-led onsite assembly training programs to acquire the necessary knowledge. Yet there is still a lack of research evidence on the effectiveness and comparative advantages of internet and offline training. This study explores the impact of various information access channels on farmers’ adoption of green production technologies in agriculture, focusing on a sample of 731 family farms located in Sichuan Province. The issue of endogeneity was addressed using the Conditional Mixed Process Estimation Method. The sample underwent a t-test and heterogeneity analysis. The findings revealed that both internet-based information access and participation in training significantly bolstered farmers’ adoption of AGPT, with the former proving to be more effective. Notably, heterogeneity was observed among farmers, differentiated by age and the number of village cadres within their family units.

## Introduction

Agriculture is the second-largest source of global greenhouse gas emissions, with only fossil fuel combustion contributing more [1]. Advancing a green transition in agricultural practices has become an essential approach to address global climate change [2]. This paradigm shift has generated significant interest in Agricultural Green Production Technologies (AGPT), which play a pivotal role in promoting sustainable agricultural development and enabling a shift towards green and low-carbon agricultural practices [3–6]. AGPT, as an innovative technology, aims to reduce environmental pollution and resource consumption during the agricultural production process while maintaining agricultural productivity. However, as an emerging technology, farmers require comprehensive information to adopt AGPT [7–10]. It has been widely emphasized that addressing new challenges related to the accessibility of information for farmers is necessary [11, 12].

Information plays a vital role in the adoption of technology [13, 14]. Farmers are typically disadvantaged regarding information access and are often mired in information poverty [15]. The primary cause of this poverty is the inadequate supply of information [16–18]. Traditional farmers have limited access to information, primarily obtaining it from their immediate surroundings [19]. The predominant channels to address this information deficit include government-organized, authoritative onsite assembly trainings; and the democratic, autonomous Internet, which depends on information technology. Most existing studies on such training suggest that this organized and authoritative approach can foster farmers’ adoption of AGPT [20, 21]. However, these findings have not led to a consistent conclusion. Some studies have found no relationship between training and technology adoption [22]. Therefore, the impact of training on farmers’ adoption of AGPT remains an unresolved question.

Secondly, with regard to information access via the Internet, the public goods characteristic of agricultural information becomes relevant [23]. In instances where farmers either do not participate in trainings or are unable to acquire the necessary information during the trainings, the Internet, with its low entry barrier, offers an alternative avenue for farmers to access agricultural information independently. The broadening of such avenues offsets the information disadvantages faced by farmers [24–26]. This compensation for information disadvantage catalyzes the adoption of new technologies by farmers. Furthermore, the Internet expedites farmers’ adoption of AGPT [26]. Internet-based agricultural extension services can significantly enhance farmers’ adoption of AGPT, such as soil-formulated fertilizer application and water conservation techniques. However, while the Internet can improve farmers’ information accessibility, it also requires an increased capacity for information processing on the part of the farmers [24, 27]. It is important to note that the information poverty experienced by farmers is not solely characterized by limited access to information but also by their inability to discern valuable information. Consequently, leveraging the Internet to facilitate farmers’ acceptance of new technologies may not be effective among vulnerable populations [28].

Hence, the effectiveness of promoting farmers’ adoption of AGPT through exclusive reliance on either training or the Internet remains a contentious issue. This study concurrently examines both channels to further elucidate their respective impacts. Additionally, there is a notable lack of scholarly research comparing the effects of these two information access channels.

This paper’s main contributions could be summarized in two primary ways. First, it incorporates two distinct information access channels—participation in training and experimental Internet use—into a unified analytical framework. This approach sheds further light on the impact of both channels on farmers’ likelihood of adopting green production technologies. It assists farmers in improving their information acquisition capabilities in a targeted manner, diversifying their information access, and effectively alleviating information poverty. Second, this study provides empirical evidence regarding the relative effectiveness of the two information access channels—the Internet and training. This evidence is poised to guide the government, relevant policymakers, and those committed to fostering the growth of green agriculture, enabling them to make more informed decisions.

## Theoretical analysis and hypotheses

Farmers, as rational investors, base their decisions on the knowledge they acquire [29]. However, they are typically entrenched in information poverty and necessitate access to information to fulfill their requirements, thereby categorizing them as a quintessential information-disadvantaged group [30]. There exists a pervasive lack of understanding among farmers regarding AGPT, an emergent technology in agricultural production. Rogers’ diffusion of innovations theory underscores that adopting innovative technology is facilitated if it caters to people’s immediate needs. Consequently, equipping farmers with pertinent information is a prerequisite for encouraging their adoption of AGPT.

Currently, the primary conduits for farmers to acquire information on AGPT are training and the Internet. From the perspective of training, it somewhat fulfills the knowledge requirements of farmers. Government-sponsored training programs, for instance, can significantly enhance farmers’ knowledge [13]. Research conducted using data collected in Yunnan Province, China, revealed a substantial increase in farmers’ knowledge following training [31]. Moreover, agricultural training was found to enhance Chinese farmers’ comprehension of green production in agriculture [32]—government-conducted training, being authoritative and targeted, augments farmers’ understanding. However, scheduled training can also induce a “peer effect,” motivating neighboring farmers to adopt AGPT [33]. A study examining the technical training of peanut growers and the peer effect of technical training demonstrated that technical training provided by neighboring farmers has a more profound impact on farmers’ pesticide usage behavior than technical training provided by individual farmers. Farmers may acquire knowledge by observing and learning from neighboring farmers who have undergone the training, thereby leading to the adoption of AGPT behavior.

While training can cater to farmers’ informational needs and facilitate their adoption of AGPT [20]. Its effectiveness in practice is subject to debate. Firstly, there exists a deficiency in the diffusion of information among farmers that have undergone training. On one hand, the dissemination of new agricultural technologies to farmers is impeded by human and financial constraints [34]. This implies that only a select group of farmers, especially large-scale farms, are targeted for technology dissemination rather than all farmers receiving agricultural training. On the other hand, a large number of studies have found that "social learning" plays a vital role in the adoption of technology [35–38]. Knowledge transfer from trained farmers to untrained farmers can facilitate technology adoption by untrained farmers [39]. However, there is no evidence to suggest that trained farmers impart the knowledge they acquire to untrained farmers [22, 40, 41], indicating that agricultural education has not effectively permeated farming households. Secondly, agricultural training programs are primarily organized by local governments. Information is disseminated to farmers in a largely unidirectional manner, with farmers seldom providing feedback on the outcomes of the training program. However, the effectiveness of the program is influenced by several factors, including the scope of the training, the technical difficulty, and the level of interactivity [42]. Farmers exhibit a preference for information that is tailored to their specific needs. Those who partake in government-led training are relegated to a passive, receptive role, and the information they acquire may not necessarily align with their requirements. Given that only a select group of farmers are targeted for technology dissemination, this form of government-led training may preclude the majority of farmers from gaining knowledge about AGPT. Moreover, the trained farmers may not receive information that caters to their needs. These factors constrain farmers’ access to pertinent AGPT-related information and influence the success rate of farmers adopting AGPT.

In contrast to training, farmers can generally independently acquire information from the Internet. With the continuous advancement of rural Internet infrastructure, the Internet has rapidly emerged as a pivotal information source for farmers [43]. It plays a substantial role in incentivizing farmers to adopt AGPT.

To commence, the advent of the Internet has empowered farmers to access information more readily and autonomously. Furthermore, the Internet has rendered information access less costly [44], providing farmers with timely and targeted information at a fraction of the cost. The Internet transcends temporal and spatial barriers, significantly amplifies farmers’ social networks, and broadens their information sources. In traditional rural societies, farmers’ access to information is predominantly restricted to their immediate surroundings [19, 45]. This Internet-based social network exposes farmers to diverse information, satisfying their inclination to learn from others’ experiences and bolstering their confidence in technology [46]. Secondly, propagandists can utilize images, films, and other media forms to depict theoretical agricultural information on the Internet, enhancing engagement and interactivity [47]. This, in turn, encourages farmers to adopt AGPT and aids in their comprehension of agricultural knowledge. Additionally, in Bandura’s social cognitive theory, viewers who observe the actions of role models and the outcomes of those actions are likely to emulate those behaviors [48]. By presenting role models, the Internet motivates farmers to adopt new behaviors.

Despite the plethora of information available on the Internet, farmers may be misled by inaccurate information or may fail to discern valuable information due to their inherent limitations [49, 50]. Nevertheless, this study posits that, despite these challenges, farmers’ access to information via the Internet is still more adept at fulfilling their informational needs and fostering their adoption behaviors towards AGPT. This assertion is predicated on the preceding analysis of the two information access channels—training and the Internet. The following theories are put forth in this paper based on the debate above: Hypothesis (H1): Farmers’ adoption of AGPT is positively impacted by accessing information on training; Hypothesis 2 (H2): Farmers’ adoption of AGPT is positively impacted by accessing information on the Internet;

Hypothesis 3 (H3): Accessing information on the Internet is more effective than accessing information on training in encouraging farmers to adopt AGPT.

## Research methodology

The empirical analysis in this paper employs the Oprobit model, a statistical instrument adept at scrutinizing dependent variables that possess multiple ordered levels, particularly when handling ordered categorical dependent variables. The dependent variable in this paper is AGPT, an ordered categorical variable, which assumes values from the set [4]. As a result, the Oprobit method was chosen for this investigation. The fundamental form of the model is as follows:

$$Y^{*}=\beta_{0}+\alpha_{1}M+\alpha_{2}P+\gamma X+\mu$$ (1)

Y* denotes the level of farmers’ adoption of AGPT, *M* denotes the level of farmers’ access to information through the Internet, *P* denotes the level of farmers’ participation in training to obtain information, *X* denotes the control variable, *β*_0_ indicates the intercept term, *α*_1_, *α*_2_, *γ* is the parameter to be evaluated, and *μ* is the residual.

For better prediction, a latent regression equation is needed based on the latent variable Y*:

$$Y^{*}=\beta_{0}+c_{1}X_{1}+c_{2}X_{2}+\cdots \cdots +c_{n}X_{n}+\varepsilon_{0}$$ (2)

Since Y* cannot be directly observed, it is necessary to set a threshold. This study uses the quartile method to select the threshold and establish the relationship between Y and Y*. The details are as follows: Since Y* cannot be obtained by direct observation, it needs to be set by the threshold value (*δ*). In this study, the quartile method is used to select the threshold value, and the relationship between Y and Y* is established as follows:

$$Y=\left\{\;\begin{array}{l} 0,\;\;0\leq Y^{*}\leq 3\\ 1,\;\;4\leq Y^{*}\leq 7\\ 2,\;\;8\leq Y^{*}\leq 11\\ 3,\;\;12\leq Y^{*}\leq 14 \end{array}\right.$$ (3)

**Fig 1:**
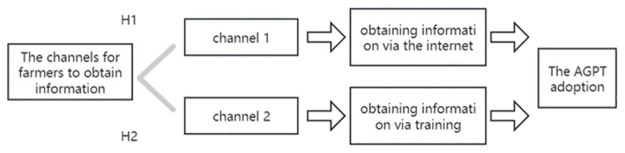
Theoretical framework.

We categorized farmers’ adoption of AGPT into the highest level of adoption (Y = 3), high level of adoption (Y = 2), low level of adoption(Y = 1), and the lowest of adoption(Y = 0) to standardize the sample. Therefore, the Oprobit model was used in this paper to estimate farmers’ adoption of AGPT. Based on the above analysis, this paper constructs the theoretical framework shown in Fig 1.

## Variable descriptions and sample sources

### Sample sources

Sichuan Province, recognized as a typical agricultural province, has seen its agricultural and rural development attract considerable attention. Concurrently, numerous villages in Sichuan Province have initiated various training programs for farmers. According to pertinent media reports, the Sichuan Provincial Government has been steadfast in promoting the development of the Internet and eco-technology in rural areas. As the penetration of training and the Internet intensifies in rural areas, farmers are becoming increasingly cognizant and are incorporating more technologies into their agricultural production. During the period from July to September 2020, the research team randomly selected 11 prefecture-level cities from three economic regions in Sichuan Province. We chose one or two well-developed towns in the selected cities for our study. The primary subjects of the research were family farms. The team researched their basic information, Internet usage, understanding of green production technology, training participation, and agricultural technology application. In our non-interventional studies, all participants were fully informed why the research was being conducted, how their data would be used and if there are any risks associated. We made sure that this study was conducted with the consent of the subjects and under the supervision of our university. The research findings were used to generate a sample of 731 research subjects.

Our research does not involve minors, and all participants have provided informed consent. Each field investigation team consists of at least two interviewers. One interviewer seeks the respondent’s consent for data collection, analysis, and publication, explaining the study’s purpose, how their data will be used, and any potential risks. The second interviewer witnesses the explicit consent process. We ensure that this research is conducted under the supervision of our university and with the subjects’ consent. We are committed to safeguarding the participants’ data and will not disclose or release personal information inappropriately. The data has been anonymized, and this study poses no risk to human subjects.

**Fig 2:**
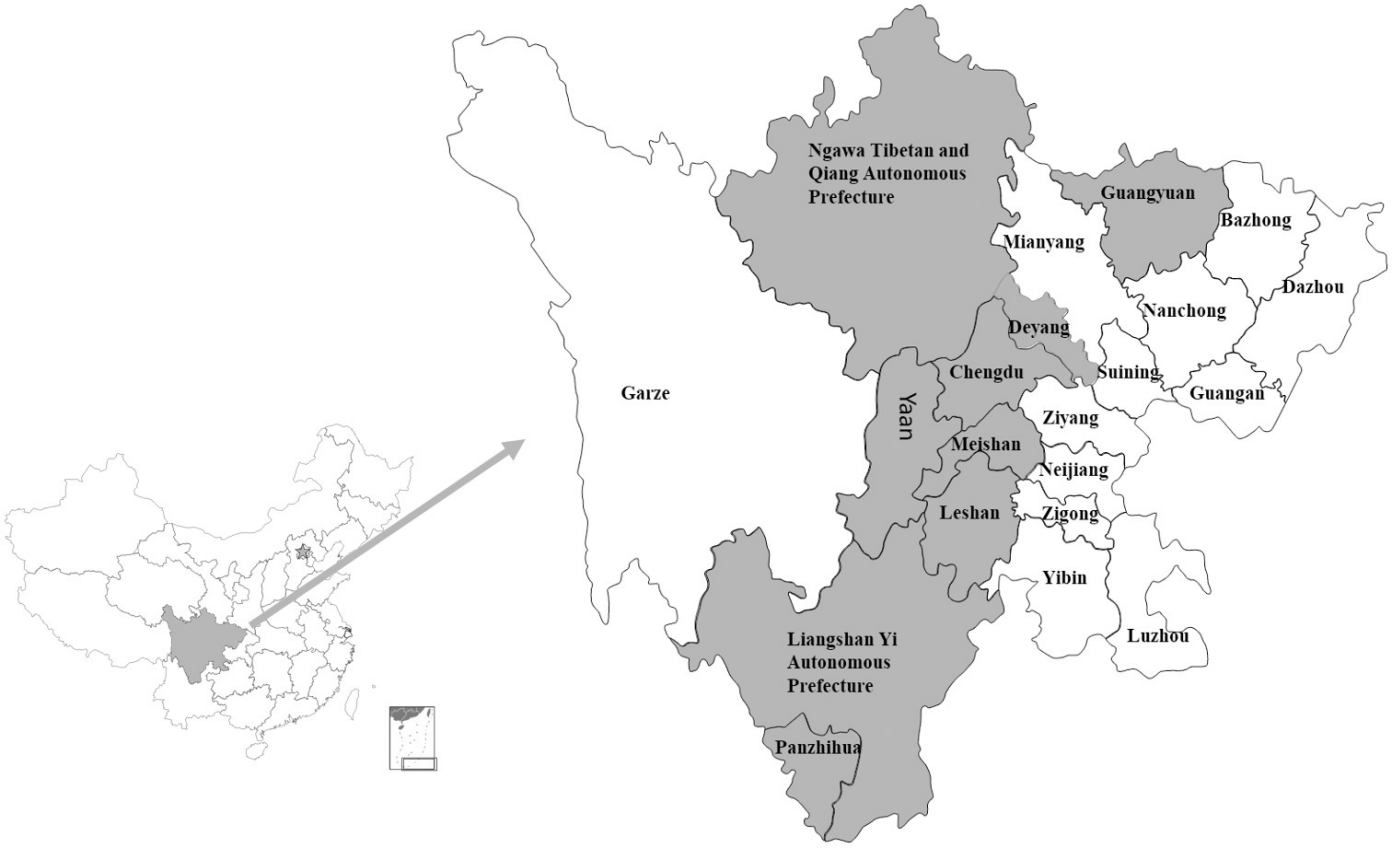
Map of the sampled area.

The map of the sampled area is shown in Fig 2. (Note: The shaded area represents the research zone).

## Descriptive statistics and variable selection

### Independent variable

1. Obtaining information via the internet. Farmers utilize the Internet to access information tailored to their specific needs. This includes six distinct aspects: direct information access, online socialization for information acquisition, and the extent of application of agriculture-related Internet platforms. These aspects are partially measured using factor dimensionality reduction.
2. Obtaining information via training. In line with the progressive implementation of the rural revitalization strategy, governments have introduced measures encompassing training and assistance to farmers in AGPT. The training in AGPT encompasses three dimensions: government-led training, enterprise-led training, and training provided by village organizations. The measurement is conducted by inquiring whether they have received training in AGPT from the government, enterprises, or village organizations. The frequency of training received is quantified based on the number of occurrences, with an annual maximum limit set at ten instances.

### Dependent variable

The AGPT adoption. Drawing upon the research data, the adoption of AGPT is classified using the quartile method, encompassing four categories: the highest level of adoption (Y = 3), high level of adoption (Y = 2), low level of adoption (Y = 1), and the lowest level of adoption (Y = 0). This classification is informed by the technology adoption model developed by Obal, Michael [51]. The adoption of AGPT by farmers is comprehensively delineated from three perspectives: strategy formulation, technology adoption, and development model. This encompasses a total of nine question items, specifically including aspects such as rural tourism and sightseeing, catering and accommodation, picking experience, and adoption and customization strategies. The results of factor dimensionality reduction are employed as the final dependent variable.

### Control variables

The heterogeneity analysis, focusing on personal situations, is grounded in existing research and draws upon the Impact of Individual Differences on Entrepreneurship Scale developed by Jo Hyejung [52]. The personal situation was gauged across four dimensions: personal characteristics, learning and skill situation, family structure, and risk preference. This encompasses a total of 13 measurement questions. Personal characteristics include aspects such as gender and age. The learning and skill situation takes into account the education level, party membership status, village cadre status, and pre-farming occupation. Family structure considers the distance to the nearest county, the highest education level among family members, and the number of cadres or party members within the family. These measurements were assessed using factor dimensionality reduction.

In this study, we performed descriptive statistics and correlation analysis on the core variables. The explanation of variable measurements and descriptive statistics are presented in Table 1. the correlation analysis is depicted in Table 2.

**Table 1:** Measurement explanation and descriptive statistics of variables.

Variables	Concept Connotation	Metrics	Obs	Std. Dev.	Min	Max	Expected direction
**Obtaining information via training**	Number of times I have participated in agricultural green production technology training organized by various sectors of society, including the government, targeting farmers	Number of agricultural trainings in which respondents actually participated a year (from 0 to 10), continuous variable	731	2.039	0	10	Positive
**Obtaining information via the internet**	Farmers get the information they need by using the Internet	The results were factor analyzed for downscaling based on respondents’ actual choices. Specific measurement questions included “Application scope of Internet platforms related to agriculture; The scope of online sales channels adopted; Join WeChat groups related to agriculture, number of QQ groups”, continuous variable	731	0.993	-1.169	5.587	Positive
**AGPT adoption**	Positive ecological impact through the adoption of AGPT	The types of AGPT actually adopted by the respondents included topics such as "recycled agriculture", "soil improvement", and "soil testing and fertilizer application". Using the quartile method, they were categorized into four levels (from 0 to 3), continuous variable	731	0.857	0	3	Undirected
**Gender**	Gender situation of interviewees	The gender of interviewees, binary variable, 0 = female, 1 = male	731	0.356	0	2	Positive
**Age**	Age of interviewees	The age of respondents, continuous variable (year)	731	9.948	0	78	Negative
**Education**	The educational level of the interviewee	1 = Elementary school and below; 2 = Junior high school; 3 = High school/vocational high school; 4 = College; 5 = Bachelor’s degree and above	731	0.986	0	5	Positive
**Political background**	The political background of the interviewee	Respondents’ identity in the village, binary variable, 0 = no, 1 = yes	731	0.428	0	1	Positive
**Village cadres**	The political identity of the interviewee (missing values are replaced by 0)	Respondents’ identity in the village, binary variable, 0 = no, 1 = yes	731	0.364	0	1	Positive
**Work experience**	Interviewee’s work situation before farming	Whether the respondents had done any other work before farming, 0 = no, 1 = yes	731	0.538	0	3	Negative
**Distance from the town**	The nearest town to the interviewee’s village	Actual distance according to respondents, continuous variable (km)	731	14.269	0.500	72	Negative
**Number of civil servant**	Work situation of interviewee’s family members within the system	Actual number of civil servants in respondents’ households, continuous variable	731	0.390	0	2	Positive
**Number of village cadres**	The identity of the interviewee’s family members in their village	Actual number village cadres in respondents’ households, continuous variable	731	0.288	0	4	Positive

**Table 2:** Correlation analysis.

**Variables**	**AGPT adoption**	**Obtaining information via the internet**	**Accessing information on training**	**Gender**	**Age**	**Education**	**Political background**	**Village cadres**	**Work experience**	**Distance from the town**	**Number of village cadres**	**Number of village cadres**
**AGPT adoption**	1.000											
**Obtaining information via the internet**	0.322	1.000										
**Obtaining information via training**	0.106	0.389	1.000									
**Gender**	0.087	0.149	0.007	1.000								
**Age**	0.045	0.070	0.066	0.062	1.000							
**Education**	-0.132	-0.109	-0.020	-0.147	0.017	1.000						
**Political background**	0.008	0.008	0.068	-0.064	0.082	0.052	1.000					
**Village cadres**	-0.252	-0.256	0.040	-0.036	-0.075	0.192	0.080	1.000				
**Work experience**	0.261	0.294	0.029	0.364	0.171	-0.230	-0.034	-0.483	1.000			
**Distance from the town**	0.103	0.085	0.075	0.130	0.408	-0.026	0.097	-0.050	0.327	1.000		
**Number of civil servant**	0.083	0.072	0.049	0.100	0.767	0.018	0.055	0.004	0.179	0.486	1.000	
**Number of village cadres**	0.015	0.262	0.240	-0.011	0.060	0.101	0.125	-0.086	0.107	0.042	0.047	1.000

The results of the Pearson correlation analysis indicate that the correlation coefficient, denoted by |r|, between the variables, is fundamentally less than 0.5. This suggests that there is no severe issue of multicollinearity, and the analysis successfully passes the validity test.

## Result

Impact of access to information on farmers’ AGPT adoption. In this paper, the farmers’ information access channels are categorized into two types: accessing the information on the Internet and obtaining information via training. The regression results show a significant positive effect between Obtaining information via the internet and AGPT adoption, which is important at a 1% level of significance. There is no statistically significant effect between obtaining information via training and AGPT adoption. This may be due to the problem of multicollinearity between access to information through the Internet and participation in training. Therefore, in Equations 5 and 6, this study took the method of excluding the two variables of obtaining information via the internet and obtaining information via training one by one to conduct Oprobit regression analysis again, and the regression results are shown in Table 3.

**Table 3:** Oprobit regression results.

Variables	AGPT adoption		
	Equation 4	Equation 5	Equation 6
**Obtaining information via training**	0.320*** (0.049)	0.333*** (0.046)	——
**Obtaining information via the internet**	0.017 (0.021)	——	0.068*** (0.010)
**Gender**	0.085 (0.119)	0.087 (0.119)	0.083 (0.115)
**Age**	-0.016*** (0.005)	-0.016*** (0.005)	-0.021*** (0.005)
**Education**	0.131** (0.052)	0.129** (0.052)	0.181*** (0.051)
**Political background**	0.060 (0.110)	0.065 (0.110)	0.038 (0.110)
**Village cadres**	0.336** (0.167)	0.331** (0.167)	0.377** (0.164)
**Work experience**	-0.195** (0.084)	-0.185** (0.083)	-0.080 (0.078)
**Distance from the town**	-0.004 (0.003)	-0.004 (0.003)	-0.005* (0.003)
**Number of civil servant**	-0.265* (0.137)	-0.259* (0.137)	-0.287** (0.134)
**Number of village cadres**	-0.337*** (0.115)	-0.333*** (0.115)	-0.214* (0.121)

Note:

*, ** and *** show statistical significance at 10%, 5%, and 1% levels, respectively. Parentheses show t-statistics.

The Equation 5 demonstrates a significant positive correlation (0.068) between mandatory information access and the adoption of green agricultural technologies. This correlation remains significant at the 1% level, even when accounting for variables such as personal characteristics, knowledge and skills, family structure, and risk preferences. This suggests that the more farmers are required to obtain information, the more likely they are to adopt green agricultural production technology, confirming Hypothesis H2. The results of this study are similar to those of previous studies. Training in agricultural cooperatives can significantly enhance members’ willingness to adopt AGPT, and this impact is particularly significant for small-scale cooperatives [20]. In addition, this study is consistent with that of a Xuiling Qian who used smallholder farmers as the study population and found that training is indeed crucial for the adoption of AGPT by farmers [21]. A possible explanation for this phenomenon is that farmers who traditionally use conventional agricultural technology are exposed to modern agricultural information by participating in training related to green agricultural production technology. Compared to farmers who have not participated in the training, they have a deeper understanding of green agricultural production technology. They are more able to appreciate the benefits of this technology. Therefore, even if information access was mandatory, farmers who had participated in the training were more likely to adopt green agricultural production technologies.

Equation 6 there is a significant positive correlation (0.333) between internet access and the adoption of green agricultural technologies. This correlation is significant at the 1% level, even when controlling for variables such as personal characteristics, learning and skill profiles, family structure, and risk preferences. This suggests that the more farmers access information on the internet, the more likely they are to adopt green agricultural production technology, thereby confirming Hypothesis H1. Therefore, the embedding of the Internet has a significant role in promoting farmers to adopt relevant green technologies, which match those observed in earlier studies. [26, 27]. Possible explanations for this phenomenon include the following: After gaining access to digital resources, farmers can find information about green agricultural production technology that aligns with their needs. They become aware of the limitations of traditional agriculture and the advantages of digital green agriculture. Moreover, the mobile internet allows them to establish wider social networks and access more external resources and assistance, which in turn promotes the adoption of green agricultural production technologies.

### Robustness tests

The Oprobit model is used as the main regression analysis model, and the Ologit model is used for the robustness test. The test results are shown in Table 4.

**Table 4:** Ologit regression result.

Variables	AGPT adoption		
	Equation 7	Equation 8	Equation 9
**Obtaining information via training**	0.556*** (0.085)	0.577*** (0.079)	——
**Obtaining information via the internet**	0.026 (0.038)	——	0.116*** (0.035)
**Gender**	-0.475* (0.271)	-0.468* (0.271)	-0.506* (0.268)
**Age**	-0.537** (0.240)	-0.530** (0.240)	-0.333 (0.238)
**Education**	-0.006 (0.005)	-0.006 (0.005)	-0.007 (0.005)
**Political background**	0.163 (0.200)	0.166 (0.200)	0.141 (0.198)
**Village cadres**	-0.027*** (0.008)	-0.026*** (0.008)	-0.035*** (0.008)
**Work experience**	0.231*** (0.089)	0.228** (0.089)	0.326*** (0.088)
**Distance from the town**	0.130 (0.194)	0.136 (0.194)	0.097 (0.193)
**Number of civil servant**	0.606* (0.310)	0.600* (0.310)	0.653** (0.306)
**Number of village cadres**	-0.355** (0.141)	-0.340** (0.139)	-0.184 (0.136)

Note:

*, ** and *** show statistical significance at 10%, 5%, and 1% levels, respectively. Parentheses show t-statistics.

The Ologit model is consistent with the Oprobit model regression results, and the findings remain robust.

### Endogeneity analysis

Endogeneity, which arises from the correlation between independent variables and disturbance terms, can lead to infeasible regression results. This paper identifies potential endogeneity between the availability of information and farmers’ adoption of green production technologies. This endogeneity could be due to directional causality and omitted variables. Farmers who adopt higher levels of ecological technology are likely to have greater autonomy and ability to access information in their agricultural production, leading to increased information access. This directional causality could result in an overestimation of the impact of information availability in the study. Additionally, the scope of the research may omit variables that can influence both the level of information availability and farmers’ adoption of green production technologies. These variables could include hard-to-measure factors such as farmers’ psychological factors and environmental factors. If the endogeneity resulting from reverse causality is more pronounced, it could lead to biased estimation results. To mitigate the endogeneity problem, this paper employs a mixed process estimation method. The results of this method are presented in Table 5.

**Table 5:** CMP test.

Variables	Stage 1		Stage 2
	Obtaining information via the internet	Accessing information on training	AGPT adoption
**Predictive values of instrumental variables (Obtaining information via the internet)**	0.618*** (0.062)	——	0.616*** (0.073)
**Predictive values of instrumental variables (Obtaining information via training)**	——	0.875*** (0.054)	0.111*** (0.039)
**Control variables**	Control	Control	Control
**LR chi2**	248.14***	279.38***	——
**Atanhrho_12**	-0.314***	-0.137	——
**Obs**	731	731	731

Note:

*, ** and *** show statistical significance at 10%, 5%, and 1% levels, respectively. Parentheses show t-statistics.

The instrumental variables selected for this study are the average level of neighborhood internet access and the average level of neighborhood participation in information-gathering training. We cluster the independent variables—internet access and training participation—and exclude individual data to derive the instrumental variables: the neighborhood average level of internet access and the neighborhood average level of participation in information-gathering training. These instrumental variables were used to test the model using the CMP test, which helped overcome the model’s endogeneity and obtain consistent estimates. The applicability of these instrumental variables lies in the fact that the level of a neighbor’s internet access and the level of a neighbor’s participation in information-gathering training do not directly influence the respondents’ adoption level of green agricultural production technologies. Instead, their primary impact is on the respondents’ level of information access.

The correlation test results, as presented in Table 5, reveal significant findings. The regression coefficients of the instrumental variable “average level of neighborhood internet access” on farmers’ adoption of Agricultural Green Production Technologies pass the significance test at the 1% level. Furthermore, atanhrho_12 significantly differs from zero at the 1% level. Consequently, this variable is endogenous and exhibits a more severe endogeneity problem, making the CMP regression results more reliable than the original regression results. On the other hand, the regression coefficient of the instrumental variable “average level of neighborhood training participation” on farmers’ adoption of green agricultural production technologies passes the significance test at the 1% level. However, atanhrho_12 does not pass the significance test. Therefore, we infer that this variable does not have a significant endogeneity problem, and the original Oprobit regression results (as per Equation 5) are deemed plausible.

### T-test

To further investigate the effects of different information access channels on farmers’ adoption of green agricultural production technologies and to discern whether significant differences exist in the effects produced by these channels, we conducted a quantitative comparison of the two channels. Prior to performing the T-test, we divided the sample into two subgroups: high and low. This division was based on whether the farmers in the sample accessed information via the Internet and through training at a level higher than the average. The groups were categorized as follows: “internet-led” (groups with high internet access and high training access), “training-led” (groups with high training access and low internet access), “dual-channel high access” (groups with high internet access and high training access), and “dual-channel low access” (groups with low internet access and low training access). Following this categorization, we conducted independent sample T-tests. This approach allowed us to understand better the role of different information access channels in promoting the adoption of green agricultural production technologies among farmers. The “Internet-high, Training-low” group refers to the subset of farmers within the 731 samples who exhibit a higher-than-average level of self-access to information via the Internet but a lower-than-average level of receiving information from trainers through agricultural green technology training. Conversely, the “Training-high, Internet-low” group refers to the subset of farmers within the 731 samples who demonstrate a lower-than-average level of self-access to information via the Internet but a higher-than-average level of receiving information from trainers through AGPT training. The results of these tests are presented in Table 6.

**Table 6:** T-test.

category	Obs	Min	Std	t	p
**High-internet high-training**	171	1.538	0.057	——	——
**High-internet low-training**	138	1.536	0.077	——	——
**low-internet high-training**	108	1.111	0.076	——	——
**low-internet low-training**	314	1.061	0.048	——	——
**diff(low-internet low-training—High-internet high-training)**	——	——	——	-6.4220	0.0000***
**diff(High-internet high-training—High-internet low-training)**	——	——	——	0.0186	0.4926
**diff(low-internet high-training—High-internet low-training)**	——	——	——	-3.8635	0.0001***

Note:

*, ** and *** show statistical significance at 10%, 5%, and 1% levels, respectively. Parentheses show t-statistics.

The results indicate significant differences among the four sample groups in terms of promoting the adoption of AGPT among farmers. The most effective promotion of AGPT adoption was observed when both the degree of internet-based information access and the degree of training-based information access were high. This suggests that the more channels’ farmers have for obtaining information, the more effectively they can promote the adoption of AGPT, underscoring the importance of information in the adoption of AGPT. Furthermore, the degree of AGPT adoption is higher in the “Internet-led” group than in the “Training-led” group, indicating that obtaining information through the Internet is a more effective method of information acquisition. A possible explanation for this is that when farmers use the internet to obtain the information they need, they have the autonomy to choose the information, allowing for a more accurate match with their needs, more effective information acquisition, and more efficient utilization of the information. Conversely, during the training process, farmers do not have the autonomy to choose the information they receive. The alignment between the information provided by the training provider and the information needed by individual farmers may be low, resulting in partially ineffective information acquired during the AGPT training process. This leads to lower utilization of the received information and less effective promotion of AGPT adoption among farmers.

### Heterogeneity analysis

This study conducts a heterogeneity analysis of the sample to validate the differences in effects across various groups. The basis for this grouping is determined by factors such as age, education, and the number of village cadres in the household. The outcomes of this analysis are presented in Table 7.

**Table 7:** Heterogeneity analysis.

Variable	Age		Education		Village cadres	
AGPT adoption	Equation 10 high	Equation 11 low	Equation 12 high	Equation 13 low	Equation 14 high	Equation 15 low
**Obtaining information via the internet**	0.344*** (0.062)	0.354*** (0.068)	0.421*** (0.072)	0.303*** (0.060)	0.278** (0.110)	0.350*** (0.051)
**Obtaining information via training**	0.107*** (0.029)	0.024 (0.030)	0.074** (0.035)	0.059** (0.026)	0.049 (0.044)	0.071*** (0.023)
**Control variables**	control	control	control	control	control	control
**Pseudo R2**	0.076	0.051	0.069	0.067	0.069	0.078
**LR chi2**	67.73***	44.49***	46.01***	75.14***	21.12***	117.52***
**Samples**	378	353	273	458	128	603

Note:

*, ** and *** show statistical significance at 10%, 5%, and 1% levels, respectively. Parentheses show heteroskedasticity robust standard errors.

To enhance the credibility of the heterogeneity analysis results, we utilized the mean of the sample variables as the basis for division. The sample was then divided into groups of low and high levels based on a specific variable. This approach ensures a more accurate and reliable analysis of the heterogeneity within the sample.

The outcomes of Equation 10 and Equation 11 reveal a significant disparity between farmers in the lower and higher age brackets regarding the impact of their training participation on their adoption of green agricultural production technologies. The degree of training participation among younger farmers positively influences their adoption of green agricultural production technologies, albeit not to a statistically significant extent. A potential explanation for this is that the training content may not align with the information needs of younger participants, resulting in less effective training and, consequently, a lower impact on the adoption of AGPT. Younger individuals, being more cognitively receptive, are more inclined to obtain the information they need through channels such as the Internet. In contrast, older farmers, who have limited ability to use digital channels and rely primarily on a single channel for information acquisition, find training to be a crucial source of information. Consequently, the impact of training participation on the adoption of AGPT is significant among this demographic. This outcome is contrary to previous research, which found training can prompt younger farmers to adopt AGPT [21]. A possible explanation for this might be that the different age divisions. They define young farmers as those under 50 years old. In any case, it has been confirmed that training has different impacts on the adoption of AGPT among farmers of different ages. This analysis underscores the importance of tailoring information delivery methods to different age group’s specific needs and capabilities.

Village cadres, serving dual roles as “government agents” and “farmers’ stewards,” possess higher cognitive abilities and stronger social networks compared to ordinary farmers [53–55]. This implies that households with village cadres are more likely to support policies and show a stronger inclination towards participating in training. However, the results from Equation 14 and Equation 15 indicate that households with a higher number of village cadres have a lesser impact on farmers’ adoption of AGPT compared to those with a lower number of village cadres. This is true for both internet-based information access and participation in training. Notably, the impact of training participation on farmers’ AGPT adoption is significantly lower in households with a high number of village cadres and is not statistically significant. A possible explanation for this could be that households with a higher number of village cadres have an overall higher cognitive ability. While they may be more willing to support policies, these farmers may also have access to more information channels, reducing the proportion of AGPT-related training in all information acquisition channels. Conversely, farmers in households with fewer village cadres have fewer options and channels, making participation in AGPT training a crucial information source for them. Therefore, the number of village cadres in a household has varying effects on farmers’ adoption of AGPT, particularly in terms of participation in training to obtain information. This analysis underscores the importance of tailoring information delivery methods to different demographic groups’ specific needs and capabilities.

The outcomes of Equation 12 and Equation 13 indicate that there is no significant difference in the effects of higher and lower educational qualifications on farmers’ adoption of green agricultural production technologies within the sample. This also accords with some previous observations, which showed that education level will not affect farmers’ adoption of Water-saving irrigation technologies and Straw-returning technology [26]. Despite educational qualification being a critical factor in assessing knowledge levels and the objective difference in information and knowledge between farmers with relatively higher and lower educational qualifications, the results of the heterogeneity analysis did not align with expectations. We posit that a possible explanation is that farmers, as rational economic actors, aim to maximize their interests when making investment decisions [56]. As long as the available information suggests to individuals with lower educational levels that the benefits of adopting the technology surpass the costs, they will be as willing to adopt the technology as farmers with higher educational levels. Therefore, under the condition of information availability, differences in educational attainment do not significantly impact the adoption of green production technologies in agriculture. Our findings further affirm their conclusions and confirm that the Internet can help farmers actively adopt AGPT.

## Conclusion and policy recommendations

The adoption of green production technology in agriculture has a positive impact on sustainable agricultural development and plays a crucial role in global environmental improvement. This study, based on a questionnaire survey of 731 farmers in Sichuan Province, classified farmers’ adoption of AGPT into four categories: high-level adoption, higher-level adoption, lower-level adoption, and low-level adoption. Using the Oprobit model, the study empirically investigated the influence of various information acquisition channels on farmers’ adoption of AGPT. The model passed the validity and robustness tests, leading to the following conclusions:

The study demonstrates that both onsite assembly training and internet-based information acquisition significantly enhance farmers’ adoption of AGPT. However, there is an endogeneity issue between internet-based information access and farmers’ AGPT adoption. The regression results were corrected using the CMP method, and the corrected results still showed that internet-based information access significantly promotes farmers’ AGPT adoption. There is no severe endogeneity problem between training-based information access and farmers’ AGPT adoption, so the basic regression results were accepted. T-test results confirmed that the group of farmers who obtain information through both the Internet and training had a significantly higher rate of AGPT adoption compared to the group of farmers who did not access information through either channel, highlighting the importance of information access. Furthermore, the study found that the effectiveness of farmers’ internet-based information access surpassed that of information access through training participation. The effectiveness of information obtains via training participation in influencing AGPT adoption varied among farmers of different ages. Both channels of information obtain differed in their effectiveness in promoting AGPT adoption based on the number of village cadres in the household. Higher age groups and households with a lower number of village officials are more suited for training to promote the adoption of green agricultural production technologies.

Based on the results of the study, we propose the following policy recommendations: first, emphasize the key role of information in the adoption of AGPT by farmers, and encourage the government, research institutes or leading agricultural enterprises to participate in the promotion of AGPT information. Second, the construction of AGPT information dissemination channels should be strengthened, combining online and offline to promote the popularization of information. The government and other information promotion subjects carry out field training, establish online technology popularization platforms, and build a wide range of information access channels through social networking sites and APPs. Attention should also be paid to the actual needs of farmers in technical training, so as to achieve a targeted supply of training information. Thirdly, improving farmers’ internal motivation to adopt AGPT. On the one hand, the government can communicate to farmers the benefits of adopting AGPT, such as providing demonstrative role models, raising farmers’ expectations of the outcome of adoption, and reducing the risk of technology adoption. on the other hand, the government can formulate a policy of financial subsidies for technology adoption to reduce farmers’ economic costs.

## Supporting information

S1 Data (XLS)
